# Effects of Locally Delivered Minocycline Microspheres in Postmenopausal Female Patients with Periodontitis: A Clinical and Microbiological Study

**DOI:** 10.3390/diagnostics12061310

**Published:** 2022-05-24

**Authors:** Georgeta-Maria Laza, Irina-Georgeta Sufaru, Maria-Alexandra Martu, Cristian Martu, Diana Antonela Diaconu-Popa, Igor Jelihovschi, Silvia Martu

**Affiliations:** 1 Department of Periodontology, Grigore T. Popa University of Medicine and Pharmacy, Universitatii Street 16, 700115 Iasi, Romania; laza.gina11@gmail.com (G.-M.L.); parodontologie1@yahoo.com (S.M.); 2 ENT Clinic Department, Grigore T. Popa University of Medicine and Pharmacy, Universitatii Street 16, 700115 Iasi, Romania; cristimartu@gmail.com; 3 Department of Oral Implantology, Removable Dentures and Technology, Grigore T. Popa University of Medicine and Pharmacy, Universitatii Street 16, 700115 Iasi, Romania; antonela.diaconu@umfiasi.ro; 4 Department of Microbiology, Grigore T. Popa University of Medicine and Pharmacy, Universitatii Street 16, 700115 Iasi, Romania; jelihovschi.igor@umfiasi.ro

**Keywords:** periodontitis, postmenopause, minocycline, microspheres, periodontal pathogens

## Abstract

The postmenopausal period, due to low hormonal concentrations, can exert a negative influence on both periodontitis and osteoporosis evolution. The present study aimed to identify potential clinical and microbiological benefic effects of locally delivered minocycline microspheres (Arestin^®^) in post-menopausal female patients with moderate and severe periodontitis. Probing depth, clinical attachment levels and bleeding on probing index, along with BANA tests for *Porphyromonas gingivalis*, *Tannerella forsythia*, and *Treponema denticola*, were performed before and at 3 months after a combined treatment of scaling, root planing (SRP), and Arestin^®^ placed in deep periodontal pockets. The association between SRP and Arestin^®^ exerted significant improvements in terms of clinical periodontal parameters, as well as significant reductions in the red complex bacteria detection.

## 1. Introduction

Periodontitis is a disease of multifactorial etiology, characterized by the destruction of periodontal support, which can result in tooth loss. The determining etiological factor is given by the oral bacterial biofilm, Gram-negative bacteria, which, following the colonization of the subgingival sites, triggers an inflammatory defense response of the host. Periodontopathogenic species include microorganisms, grouped by Socransky in color-coded complexes, in which the red complex, responsible for advanced forms of periodontitis, includes *Porphyromonas gingivalis, Tannerella forsythia*, and *Treponema denticola* [1]. The treatment of periodontitis includes the etiological stage, based on scaling and root planing (SRP), which may be associated with additional antibacterial and immunomodulatory treatments [2].

It seems, however, that since 1998, when Socransky postulated the principles of bacterial pathogenicity, a number of discoveries have called into question the exact conditions of bacterial pathogenicity. Microorganisms in the red complex have also been found in periodontally healthy patients; thus, it was postulated that commensal bacteria become pathogenic due to host changes [3]. These changes include inflammatory conditions and immune responses that can generate a more favorable environment for bacterial proliferation, leading to an increased risk of disease onset and progression [4]. 

Based on the current knowledge and understanding of periodontal diseases, periodontitis can be classified in 4 degrees of severity (mild, moderate, severe, and very severe), as well as, based on the degree of progression, in 3 grades (slow, moderate, and rapid) [5]. What must be emphasized is that the treatment plan of the patient should not be based only on the statement of the stage and grade; a complex and comprehensive diagnosis is required. 

It is in this area of discussion that the role of local and systemic risk factors for periodontal disease is involved. A number of systemic diseases and conditions are thought to influence the ability of the human body to respond with good efficiency to the aggression of periopathogenic bacteria. These include diabetes, rheumatoid arthritis, atherosclerosis, obesity, or even physiological hormonal changes which normally occur throughout life.

Postmenopause is a term to describe the post-menopausal stage. In the postmenopausal period, menstruation has disappeared for more than 12 consecutive months. Menopausal symptoms, which may include hot flashes and night sweats, vaginal dryness and sexual discomfort, depression, insomnia, changes in sexual appetite, dry skin, weight changes, hair loss, or urinary incontinence, may become milder or may disappear completely. However, some people continue to experience menopausal symptoms for a decade or more after menopause [6]. Importantly, the menopausal and postmenopausal periods are characterized by a deficiency of sex hormones, estrogen and progesterone, hormones that, in normal concentrations, have an osteo-protective role. Bone metabolism is influenced by the menopause onset, with subsequent estrogen deficiency altering the rate of bone loss [7].

Hormone deficiency can lead to osteopenia and later, osteoporosis. Of course, a number of other factors have been associated with the risk of developing osteoporosis, such as: age, the phase of involution of bone mass that occurs after adulthood between 35 and 40 years, accelerated bone loss in both sexes; genetics, with a decisive role in regulating bone density, along with skeletal geometry and bone turnover; nutrition, with a deficit in vitamins and minerals; behavioral issues, including sedentary lifestyle, smoking, or use of other harmful substances [8].

Moreover, the role of the intestinal microbiota in the onset and evolution of osteoporosis was also emphasized. Proteobacteria abundance negatively correlated with bone mass [9]. Das et al. [10] found a higher abundance of *Actinomyces*, *Eggerthella*, and *Clostridium* Cluster XlVa in subjects with osteoporosis than in systemically healthy people. Xu et al. [11] observed specific changes in the intestinal microbiota in patients with osteoporosis, an increase in the total amount of intestinal bacteria, with a prevalence of *Bacteroidetes* over *Firmicutes* in the osteoporosis group. A study by He et al. showed negative associations of *Blautia* and *Fusicatenibacter* abundance with L-lysine enriched by osteopenia, while positive associations were found between *Escherichia*/*Shigella*, *Enterobacter*, and *Citrobacter* and L-threonate. *Blautia* positively correlated with lumbar spine bone mineral density (BMD), while levulinic acid and N-acetylmannosamine negatively correlated with lumbar spine BMD and total hip BMD [12].

Several studies show that postmenopausal women with osteoporosis have a low bone mineral density and tend to have a greater loss of periodontal attachment. The skeletal demineralization in osteoporosis may also affect the resistance of the alveolar bones to the inflammatory destruction of periodontitis, along with a number of inflammatory and microbiological interactions, which relate osteoporosis to periodontal disease [13,14].

In the postmenopausal stage, due to the decrease in estrogen, there is a noticeable change in the production of various growth factors and reduced osteoblastic activity, leading to an acceleration of destruction and a decrease in bone formation, with harmful effects because the loss of attachment periodontal disease is increased [15].

The main aim of our study was to investigate the effects of local therapy with minocycline microspheres on species of the Socransky red complex (*P. gingivalis*, *T. forsythia,* and *T. denticola*) in postmenopausal female patients with moderate and severe periodontitis, starting from the hypothesis that this combined therapy will decrease the bacterial species prevalence in deep periodontal pockets.

## 2. Materials and Methods

The study included post-menopausal female subjects (no menstrual bleeding for at least 12 months), diagnosed with periodontitis. A power analysis was performed for CAL as a parameter, estimating a 5% reduction, with an 80% power and alpha 0.05, generating a result of 51 subjects in the group. We also estimated a 20% rate of abandonment. Therefore, the study was conducted on 62 subjects, in the Periodontal Clinic of the Faculty of Dental Medicine, “Grigore T. Popa” University of Medicine and Pharmacy. The exclusion criteria were represented by: smoking, systemic diseases other than osteoporosis, history of periodontal treatment or antiseptic/antibiotic/anti-inflammatory drugs in the last 3 months, hormone replacement therapy, or use of bisphosphonates. 

The patients were informed regarding the study protocol and the signed informed consent was obtained from each subject. The study was conducted according to the Helsinki Declaration norms and was approved by the Ethics Committee, Approval Form/25 May 2020.

Each subject followed a dual energy X-ray absorptiometry test (DEXA) hip examination at baseline, in order to evaluate bone density, with the T-score interpretation as follows: normal (between 1 and −1), osteopenia (between −1 and −2.5), and osteoporosis (−2.5 or lower).

All the subjects followed a comprehensive clinical dental examination which included the measurements of the following periodontal parameters: probing depth (PD), clinical periodontal attachment loss (CAL), and bleeding on probing index (BOP). The measurements were performed by two experienced and calibrated examiners, with an agreement of 99.20%, using the periodontal probe (Williams Probe PQW, Hu Friedy Mfg. Co., LLC, Des Plaines, IL, USA). The diagnosis of periodontitis stages 2–3 was based on the current periodontitis classification: CAL higher than 3 mm, PD higher than 5 mm [5]. 

Full-mouth ultrasonic scaling (Woodpecker UDS-A-LED, Guilin Woodpecker Medical Instruments Co. LTD, Guangxi, China) and root planing (Gracey curettes, Hu Friedy Mfg. Co., LLC, Des Plaines, IL, USA) were performed in one session for each subject. In addition, minocycline HCl microspheres 1 mg, incorporated into a bioresorbable polymer, poly-glycolide-co-dl-lactide (PGLA) (Arestin^®^, OraPharma, Bausch Health US, LLC, Bridgewater, NJ, USA), were placed in periodontal pockets. The antibiotic comes as a dry powder, packaged in a unit-dose cartridge, which is inserted into a spring-loaded mechanism. After isolating and gently drying the recipient site, the product was slowly injected into the periodontal pocket.

Oral hygiene instructions were given in terms of toothbrushing and dental flossing, and the patients were required to avoid the use of antiseptics, antibiotics, or anti-inflammatory drugs throughout the study period.

The BANA test (N-benzoyl-dl-arginine-beta-naphtylamide) (PerioScan, Oral B, Belmont, CA, USA) was performed in order to detect the presence of *P. gingivalis, T. forsythia,* and *T. denticola*. This qualitative colorimetric test was performed chair-side, according to the manufacturer’s indications. The sites were isolated and gently dried; supragingival plaque was removed, and subgingival plaque samples were collected with a sterile curette and placed on the test paper. The results were analyzed according to the following scores: 0 (absence, no staining), 1 (weak presence, small traces of color), and 2 (presence, dark color). The total number of tested sites was 2184.

The clinical measurements and BANA tests were performed at baseline and repeated after 3 months.

All the data were registered for each subject and statistically analyzed (Microsoft Excel 2021 and Wizard 2 for Mac, ^®^Evan Miller). A Shapiro–Wilk test was conducted as a normality test; and normally distributed values were compared with a paired *t*-Test, while for non-normally distributed values we used a Mann–Whitney U test. Pearson’s correlation test was used to measure the linear correlations.

## 3. Results

The study was conducted on 62 post-menopausal female subjects with a mean age of 59.98 ± 4.02 years old. At the 3-month evaluation, we observed significant reductions of probing depth (from 6.05 ± 0.36 mm to 5.12 ± 0.33 mm, *p* < 0.001), clinical attachment loss (from 5.11 ± 0.59 mm to 4.45 ± 0.57 mm, *p* < 0.001) and bleeding on probing (from 78.19 ± 5.00 to 11.54 ± 2.78, *p* < 0.001) (Table 1).

In addition, at 3 months, all 3 detected pathogens showed significant reductions: from 84.78 ± 6.74 at baseline to 10.75 ± 3.55 for *P. gingivalis* (*p* < 0.001), from 61.91 ± 13.40 to 8.44 ± 2.01 for *T. forsythia* (*p* < 0.001), and from 39.72 ± 6.50 at baseline to 7.53 ± 1.20 after 3 months for *T. denticola* (*p* < 0.001) (Table 2).

After the DEXA T-score analysis, we observed that none of the patients presented normal density values (between 1 and −1); 87.09% of the subjects showed values between −1 and −2.5 and 12.91% presented values lower than −2.5.

**Figure 1:**
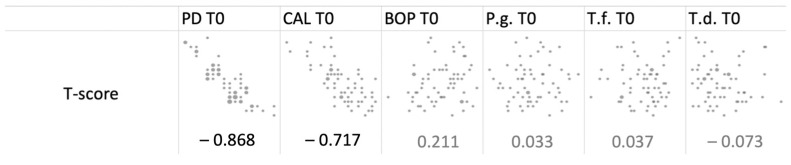
Correlation test between T-score and clinical and microbiological data at baseline (T0). PD: probing depth; CAL: clinical attachment loss; BOP: bleeding on probing; P.g.: *Porphyromonas gingivalis*; T.f.: *Tannerella forsythia*; T.d.: *Treponema denticola*.

T-score negatively correlated to the probing depth and to clinical attachment loss at baseline (−0.868 and −0.717, respectively) (as expressed in bold in Figure 1).

Regarding the correlations between clinical and microbiological findings, the only correlation we could observe at baseline was a positive one between PD and CAL (r = 0.726, *p* = 0.039) and a negative correlation between *T. forsythia* and *T. denticola* (r = −0.263, *p* < 0.001) (Table 3). At 3 months, the only correlation that was observed was between PD and CAL (r = 0.686, *p* < 0.001) (Table 4).

When analyzing the correlations between T0–T1 (Δ values) differences, we observed positive correlations between the CAL decrease and PD decrease (r = 0.347, *p* = 0.006), as well as between the CAL decrease and BOP decrease (r = 0.275, *p* = 0.030). We also found a negative correlation between *T. denticola* and *T. forsythia* decreases (r = −0.264, *p* = 0.038) (Table 5).

## 4. Discussion

The present study investigated the effects on clinical periodontal parameters and red complex periopathogens of locally delivered microspheres of minocycline HCl in post-menopausal female patients with moderate and severe periodontitis. Moreover, we also assessed the DEXA T-score at baseline, in order to provide data related to the bone density. Our T-score analysis generated a rather impressive image of low bone density values. None of the examined patients had normal bone density and a high percentage exhibited osteopenia (87.09%). There is plenty of literature data relating periodontitis to osteopenia/osteoporosis. A study conducted by Ayed et al. [16] on osteoporosis and healthy subjects observed that osteoporosis had a direct effect on the rate of progression of periodontitis that may be related to osteoporotic alveolar bone and/or changes in the subgingival environment. Alveolar bone density was significantly lower in osteoporosis patients than in healthy subjects.

In a cross-sectional study of 1329 postmenopausal women, systemic bone density was measured in the spine, hip, forearm, and whole body by DEXA [17]. The strongest associations were found between systemic bone density and CAL. Our findings are in accordance with these results, as T-score was negatively correlated to probing depth and clinical attachment loss at baseline. Contrary, in another study, T-score had a stronger correlation to the gingival bleeding index than to PD or CAL [18].

Having in mind these particular conditions of high risk, different methods of periodontal treatment have been proposed. Besides SRP, which remains the gold standard therapy in every periodontitis case, adjunctive treatments using antibiotics, antiseptics, or photodynamic therapy have been used. In one of our previous research projects, which focused on the modulation of the hosts’ inflammatory response, we found that sub-antimicrobial doses of doxycycline (Periostat^®^) exerted beneficial clinical effects in patients with osteoporosis, especially in deep periodontal pockets [19].

The general disadvantage when using oral administration of antibiotics is generated by the potential side effects, while the concentrations in periodontal pockets might not reach the optimal value. In addition to kidney or liver toxicity, systemic antibiotics can lead to severe dysbiosis, with the development of resistant microorganisms. Therefore, locally delivered antiseptic or antibiotic substances seem to be preferred in certain cases [20]. The most important issue related to local instillations or gel applications involves the high risk of being washed by the saliva flow. 

Over the years, new products have been developed in order to counteract this problem, including systems with a controlled time of drug release. Arestin^®^ is a product with slow-release minocycline included in PLGA-based microspheres. Minocycline is part of the tetracyclines group of antibiotics. It is a broad-spectrum antibiotic, with good anti-infectious effects on both Gram-positive and Gram-negative bacteria. Moreover, minocycline also proved anti-inflammatory, immunomodulatory, and antioxidant effects [21]. Minocycline intake at doses of 100–200 mg/day for 7–14 days demonstrated its benefits in reducing periodontitis progression and promoting periodontal tissue healing [22]. On the other hand, PLGA microspheres are a suitable drug carrier, are biodegradable, and are already used in many medical devices [23]. The polymer microspheres will progressively resorb and minocycline is slowly released into the periodontal pocket for at least two weeks [24].

There are various investigations on the positive effects of locally delivered minocycline but, to our knowledge, this is the first study that focused on post-menopausal patients. Van Dyke et al. [25] observed more significant reductions in PD and CAL in patients who followed SRP + Arestin^®^ than in patients with SRP alone. Our results support these findings, with reductions of PD and CAL of 0.93 ± 0.18 and 0.66 ± 0.17 mm, respectively (*p* < 0.001 for both variables). In another study, Chackartchi et al. [26] investigated the clinical benefits of using either a chlorhexidine chip or minocycline microspheres in patients with chronic periodontitis during supportive periodontal treatment; PD, CAL, and BOP were assessed at baseline, 3, 6, and 12 months. Both treatments induced significant periodontal improvements, also reducing the need-for-surgery index. We found this aspect particularly important, due to the high patient costs and discomfort associated to surgical procedures.

In our study, minocycline significantly reduced the prevalence of red complex bacteria at the 3 months evaluation, when compared to baseline (*p* < 0.001). Goodson et al. [27] focused on the antimicrobial effect of minocycline microspheres associated to scaling and root planing. This particular combined treatment exerted significantly higher reductions of red complex bacteria counts, along with improvements in PD, CAL, and BOP. In another study, patients with periimplantitis who followed SRP + locally delivered minocycline microspheres had low bacterial levels of *T. forsythia*, *P. gingivalis,* and *T. denticola* over a 12-month period [28]. 

Teles et al. [29] investigated minocycline-resistant species percentage and taxonomy in saliva and subgingival plaque samples before and after minocycline microspheres application; the authors observed the similar clinical improvements but, more importantly, antibiotic resistance by *Aggregatibacter actinomycetemcomitans*, *T. forsythia*, and *P. gingivalis* was either absent or infrequent.

Contrary to these results, Killeen et al. [30] and Tabenski et al. [31] found no additional advantages of locally delivered minocycline versus SRP alone in deep periodontal pockets, in terms of clinical, immunological, and microbiological findings.

In an observational cross-sectional study that analyzed the oral microbiota of postmenopausal women with and without osteoporosis, the authors found that 79% of patients with periodontitis had osteoporosis but no differences were found in the quality and quantity of the investigated bacteria [32]. An interesting aspect observed in our study is given by the negative correlation between *T. forsythia* and *T. denticola* in postmenopausal women. We consider that these aspects require further investigations, on levels beyond clinical research.

Our study has a few limitations, nevertheless. First of all, our study is not a controlled one. Further investigations are required in order to support the efficiency of locally delivered minocycline as adjunctive to the non-surgical periodontal therapy versus SRP alone in patients with low bone mineral density. In addition, supplementary research is necessary to compare the effects of this particular treatment with other local drug adjunctive therapies, such as photodynamic laser/LED assisted periodontal treatment.

Second, the microbiological test we used is a qualitative one, conducted in the dental clinic. Quantitative analysis, such as polymerase chain reaction test, might offer a more accurate image for the benefit of locally delivered minocycline in terms of bacterial charge. Moreover, we consider that molecular investigations on proinflammatory cytokines and bone destruction markers are necessary in order to provide a more in-deep image of the underlying mechanisms and actions of minocycline in postmenopausal patients with periodontitis.

Nonetheless, we consider this study as able to open new directions of research, in order to clarify the intriguing and inter-twined relationship between periodontitis, menopause, and low BMD, as well as to identify and establish clear protocols for the periodontal treatment in these particular conditions.

## 5. Conclusions

Within the limitations of the present study, our hypothesis was confirmed. The association of scaling and root planing with locally delivered microspheres of minocycline generated significant improvements in periodontal tissue loss, quantified by probing depth and clinical attachment loss, as well as in gingival inflammation, measured by bleeding on probing.

This particular adjunctive therapy also exerted significant reductions in the prevalence of the red complex periodontal pathogens in postmenopausal female patients with moderate and severe periodontitis.

Therefore, we observed that locally delivered microspheres of minocycline might represent an efficient adjunctive periodontal therapy in postmenopausal subjects with periodontitis and low bone mineral density.

## Figures and Tables

**Table 1:** Changes in periodontal clinical parameters from T0 to T1.

	Baseline (T0)	At 3 Months (T1)
PD (mm)	6.05 ± 0.36	5.12 ± 0.33 *
CAL (mm)	5.11 ± 0.59	4.45 ± 0.57 *
BOP	78.19 ± 5.00	11.54 ± 2.78 *

PD: probing depth; CAL: clinical attachment loss; BOP: bleeding on probing. Values are expressed as mean ± standard deviation. * *p* < 0.001.

**Table 2:** Changes in periodontal pathogens prevalence from T0 to T1.

	Baseline (T0)	At 3 Months (T1)
*Porphyromonas gingivalis*	84.78 ± 6.74	10.75 ± 3.55 *
*Tannerella forsythia*	61.91 ± 13.40	8.44 ± 2.01 *
*Treponema denticola*	39.72 ± 6.50	7.53 ± 1.20 *

Values are expressed as mean ± standard deviation. * *p* < 0.001.

**Table 3:** Correlation coefficients (r) at baseline (T0).

	PD T0	CAL T0	BOP T0	P.g. T0	T.f. T0	T.d. T0
PD T0	0	0.000000 *	0.152733	0.280677	0.376881	0.792804
CAL T0		0	0.165768	0.487371	0.972045	0.399207
BOP T0			0	0.394453	0.433201	0.300487
P.g. T0				0	0.842481	0.113247
T.f. T0					0	0.039031 *
T.d. T0						0

PD: probing depth; CAL: clinical attachment loss; BOP: bleeding on probing; P.g.: *Porphyromonas gingivalis*; T.f.: *Tannerella forsythia*; T.d.: *Treponema denticola*; * statistical significance.

**Table 4:** Correlation coefficients (r) at 3 months (T1).

	PD T1	CAL T1	BOP T1	P.g. T1	T.f. T1	T.d. T1
PD T1	0	0.000000 *	0.971876	0.183483	0.217513	0.166559
CAL T1		0	0.477021	0.623212	0.886567	0.450642
BOP T1			0	0.748079	0.253379	0.308013
P.g. T1				0	0.307023	0.286488
T.f. T1					0	0.828705
T.d. T1						0

PD: probing depth; CAL: clinical attachment loss; BOP: bleeding on probing; P.g.: *Porphyromonas gingivalis*; T.f.: *Tannerella forsythia*; T.d.: *Treponema denticola*; * statistical significance.

**Table 5:** Correlation coefficients (r) of differences in values (Δ) T0 vs. T1.

	ΔPD	ΔCAL	ΔBOP	ΔP.g.	ΔT.f.	ΔT.d.
ΔPD	0	0.005746 *	0.426438	0.907375	0.763760	0.683836
ΔCAL		0	0.030416 *	0.828127	0.612912	0.345614
ΔBOP			0	0.250398	0.504814	0.188132
ΔP.g.				0	0.320476	0.297842
ΔT.f.					0	0.038469 *
ΔT.d.						0

PD: probing depth; CAL: clinical attachment loss; BOP: bleeding on probing; P.g.: *Porphyromonas gingivalis*; T.f.: *Tannerella forsythia*; T.d.: *Treponema denticola*; * statistical significance.

## Data Availability

The data used to support the findings of this study are available from the corresponding author upon reasonable request.
